# Practical adoption of state-of-the-art hiPSC-cardiomyocyte differentiation techniques

**DOI:** 10.1371/journal.pone.0230001

**Published:** 2020-03-10

**Authors:** Cassady E. Rupert, Chinedu Irofuala, Kareen L. K. Coulombe

**Affiliations:** Center for Biomedical Engineering, School of Engineering and Division of Biology and Medicine, Brown University, Providence, RI, United States of America; University of Louisville, UNITED STATES

## Abstract

Human induced pluripotent stem cell (hiPSC)-derived cardiomyocytes are a valuable resource for cardiac therapeutic development; however, generation of these cells in large numbers and high purity is a limitation in widespread adoption. Here, design of experiments (DOE) is used to investigate the cardiac differentiation space of three hiPSC lines when varying CHIR99027 concentration and cell seeding density, and a novel image analysis is developed to evaluate plate coverage when initiating differentiation. Metabolic selection via lactate purifies hiPSC-cardiomyocyte populations, and the bioenergetic phenotype and engineered tissue mechanics of purified and unpurified hiPSC-cardiomyocytes are compared. Findings demonstrate that when initiating differentiation one day after hiPSC plating, low (3 μM) Chiron and 72 x 10^3^ cells/cm^2^ seeding density result in peak cardiac purity (50–90%) for all three hiPSC lines. Our results confirm that metabolic selection with lactate shifts hiPSC-cardiomyocyte metabolism towards oxidative phosphorylation, but this more “mature” metabolic phenotype does not by itself result in a more mature contractile phenotype in engineered cardiac tissues at one week of culture in 3D tissues. This study provides widely adaptable methods including novel image analysis code and parameters for refining hiPSC-cardiomyocyte differentiation and describes the practical implications of metabolic selection of cardiomyocytes for downstream tissue engineering applications.

## Introduction

Human induced pluripotent stem cell (hiPSC)-derived cardiomyocytes are a promising cell source for cardiac regeneration, therapeutic development, and disease modeling. These cells, however, are difficult and costly to generate, which limits their accessibility. In order for the full potential of hiPSC-cardiomyocytes to be realized, careful and widely adaptable characterization of differentiation and purification techniques must be undertaken and made available.

Though cardiomyocyte differentiation from hiPSCs has advanced significantly, there remain challenges to its reliability and reproducibility both within and across research groups. Differentiation was first described in the spontaneous differentiation of human embryonic stem cells (hESCs) in embryoid bodies [1] and evolved rapidly to a monolayer culture method, relying on the application of recombinant human proteins to module activin/nodal and BMP signaling to mimic embryonic heart development [2]. More recently, small molecules have been used to modulate the biphasic Wnt signaling pathway that is both necessary and sufficient for cardiac specification in a chemically defined differentiation process [3,4]. While these advances have made cardiomyocyte differentiation increasingly feasible and amenable to clinical translation, they have arisen in parallel with the field’s increasing use of human induced pluripotent rather than embryonic stem cells [5]. HiPSCs have a less stable pluripotent state [6] which may result in increased heterogeneity from directed cardiac differentiation compared to hESCs.

There are several factors particularly critical for successful generation of hiPSC-cardiomyocytes during small-molecule differentiation. Cardiac differentiation is initiated with the application of a GSK3 inhibitor to activate Wnt signaling [7], which has been previously optimized at a concentration of 6 μM when using CHIR99027 (Chiron) by different groups [3,4,8–10]. The concentration of GSK3 inhibitor required to initiate the mesodermal lineage depends most significantly on the proportion of induced pluripotent or embryonic stem cells (here on referred to as “hPSCs”) in the S/G2/M stage of the cell cycle [11]. This proportion, in turn, depends primarily on cell density, colony size, and time in culture [12]. These relationships have been uncovered using endpoint analysis of cardiomyocyte purity resulting from differentiation. For practical application, methods to estimate cell cycle state and choose GSK3 inhibitor concentration prior to the initiation of cardiac differentiation must be developed.

HiPSC-cardiomyocyte generation and purification are nuanced processes that can be prohibitively difficult and costly for widespread adoption. As the use of small-molecule differentiation for hPSC-cardiomyocyte production becomes the standard in cardiovascular engineering, rigorous, repeatable techniques for characterizing and optimizing differentiation conditions are invaluable. In this study, we evaluate heterogeneity of cardiac differentiation within the experimental space of published protocols [10], provide tools to optimize cardiac purity, and investigate shortcomings of these processes. We use a design of experiments (DOE) approach with response surface modeling to evaluate cardiac differentiation conditions across multiple hiPSC lines; develop an image analysis pipeline to identify the range of hiPSC densities early after plating in which directed differentiation is successful; and demonstrate that, although metabolic selection matures hiPSC-cardiomyocyte bioenergetic phenotype in two-dimensional culture, the process alone does not improve engineered cardiac tissue function. These findings provide a valuable resource for groups already performing cardiac differentiation as well as those new to the field. We provide useful tools to standardize differentiation and important insights into how hiPSC-cardiomyocyte metabolic purification affects cell phenotype.

## Materials and methods

### Stem cell culture

Three human induced pluripotent stem cell (hiPSC) lines were used: GiPSC, NCRM-5, WTC11 (Table 1). Cells were cultured on 10 cm tissue culture plastic dishes (Fisher) coated with truncated human vitronectin (VTN-N, ThermoFisher) in Essential 8 medium (E8^™^, ThermoFisher). Medium was changed daily, and cells were passaged every 4 to 5 days at a 1:10 split ratio using in-house versine (DPBS, ThermoFisher, with 0.5 mM EDTA and 1.1 mM dextrose, Sigma-Aldrich).

**Table 1:** Human induced pluripotent stem cell lines.

Common name	Line name	Source	Donor Source	Reprogramming method
GiPSC	Gibco Episomal hiPSC Line	ThermoFisher Scientific (Catalog #A18944)	Female, CD34+ cord blood	Episomal
NCRM-5	NCRM-5	NIH Center for Regenerative Medicine	Male, CD34+ cord blood	Episomal
WTC11	WTC11, GM25256	Gladstone Institute of Cardiovascular Disease, UCSF	Male, dermal fibroblasts	Episomal

### Cardiomyocyte differentiation and purification

HiPSC-cardiomyocytes were differentiated as previously described [13]. In brief, hiPSCs were harvested in versene, triturated into small colonies, counted, and seeded on (1:60 dilution) Matrigel (Corning)-coated 6 or 24 well plates in E8^™^ with 5 μM Y-27632 (ROCK inhibitor, Fisher). Medium was changed daily until initiation of differentiation (day 0), when cells were switched to CDM3 medium [8] and treated sequentially with 6 μM (unless otherwise indicated) of GSK3 inhibitor CHIR 99021 (Chiron, Fisher), then 5 μM inhibitor of Wnt protein 2 (IWP2, Fisher)[10]. Cells were switched to RPMI 1640 medium with B27 supplement with insulin (RPMI/B27) at day 9 of differentiation. Both unpurified control cells and cells undergoing lactate purification were replated at 5–6 x10^6^ cells/cm^2^ on Matrigel-coated 6 well plates after widespread appearance of beating in RPMI/B27 (10–13 days differentiation) and were not fed for 4 days (as a starvation period). Cells for purification were then cultured for four days in lactate purification medium (LPM: glucose-free DMEM + 4mM L-glutamine, 1X non-essential amino acids, 1X GlutaMAX^™^, and 4 mM lactate) [14] and medium refreshed after 2 days. Unpurified control cells were maintained in RPMI/B27 during this period and fed on the same schedule. All hiPSC-cardiomyocytes were maintained in RPMI/B27 until use between 14 days (for differentiation optimization and image analysis studies) and 24 days (for experiments involving lactate-purified samples) of differentiation.

### Adult human cardiac fibroblast culture

Healthy adult human ventricular cardiac fibroblasts (hCFs; PromoCell) were maintained on tissue culture plastic in DMEM/F12 with 10% FBS and 1% penicillin-streptomycin (ThermoFisher). Cells were passaged upon reaching confluency in versene with 0.05% trypsin (ThermoFisher). HCFs were used in engineered tissues at passage 4 (p4).

### Engineered cardiac tissue formation

Engineered cardiac tissues (ECTs) were formed as previously described.[15] Briefly, hiPSC-cardiomyocytes were harvested at day 24 of differentiation and combined with 1.25 mg/mL rat-tail collagen-1 at 1x10^6^ cells per tissue + 5% hCFs. ECTs were cultured under static stress [16] in RPMI/B27 with 1% penicillin-streptomycin and field-stimulated at a 1 Hz (IonOptix C-pace EP). ECTs were cultured for one week before further experiments.

### Metabolic characterization

Unpurified and lactate purified hiPSC-cardiomyocytes were harvested at day 24 of differentiation and seeded at 40 x 10^3^ cells/well of a Matrigel-coated XFe96 cell culture microplate (Agilent). On day 27, cells were washed three times and then fed 175 μL/well assay media: XF Base Medium (Agilent), 4 mM GlutaMAX^™^, 1 mM Sodium Pyruvate (ThermoFisher), and 25 mM glucose (Sigma-Aldrich) and incubated for 90 minutes at 0% CO_2_ and 37°C. Basal oxygen consumption rate (OCR) and extracellular acidification rate (ECAR) measurements were made with a Seahorse XFe96 Extracellular Flux Analyzer (Agilent).

### Image analysis

Analysis of phase contrast images taken on day 0 of differentiation with a Canon EOS 6D camera mounted on a Nikon Eclipse TS100 microscope was performed using MATLAB (R2019a, MathWorks). Each image was divided into 16 subimages, individually thresholded using Otsu’s method, and binarized to calculate a fraction of well coverage. Code is available through the Brown University Dataverse in the Harvard Dataverse, DOI: https://doi.org/10.7910/DVN/KWI737.

### Flow cytometry

Cardiac purity was determined for each hiPSC-cardiomyocyte differentiation run. Cells were fixed in 4% paraformaldehyde and labeled for the definitive cardiac marker cardiac troponin T (cTnT, antibody Clone 13–11, 2 μg/mL; ThermoFisher). Samples were analyzed with a FACSAria IIIu cell sorter (BD Biosciences) and 10,000 events were recorded per sample. Flow cytometry data was analyzed used opensource Flowing Software (Turku Centre for Biotechnology), and gates were set based on isotype antibody controls.

### Mechanics testing

Engineered cardiac tissues (ECTs) underwent mechanical analysis after one week of culture as previously described [15]. Tissues were mounted on one side to a 5 mN load cell and on the other to a high-speed length controller (Aurora Scientific). ECTs were bathed in continuously perfused Tyrode’s solution with 1.8 mM Ca^2+^ between 32 and 35°C. Tissue diameter was measured at relaxed length (L_0_) and cross-sectional area (CSA) shape was assumed to be elliptical with H = 0.5W for normalization. Tissues were field stimulated at 1 Hz and stretched from L_0_ to 130% of L_0_ in 5% length steps, with twitch contractile force measured at each step. At 130% L_0_, ECTs were electrically paced from 1 Hz to 4 Hz at 0.5 Hz steps.

### Statistics

Quadratic models for differentiation optimization experiments were calculated using DOE, and goodness of fit was analyzed with ANOVA. Statistical analyses of the obtained data in lactate purification studies were performed using two-tailed unequal variance Student’s *t*-tests. Significance was considered as P < 0.05. Mean and standard error of the mean were plotted using Graphpad Prism. Sample size (n) is indicated in the text and each figure.

## Results

### Seeding density and Chiron concentration strongly influence cardiac differentiation purity

**Fig 1:**
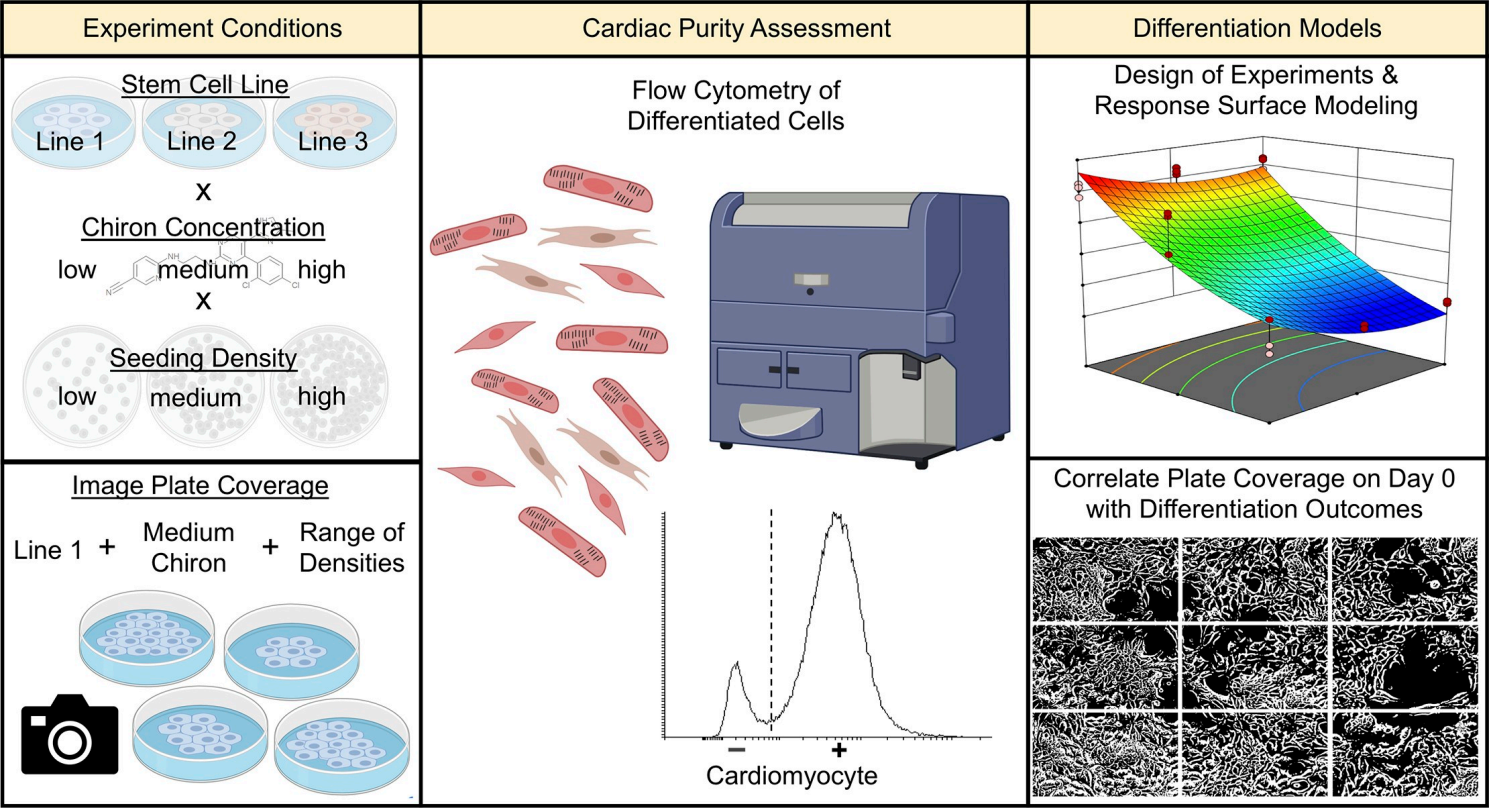
Schematic of differentiation optimization experiments. Left column: Design of experiments (DOE) studies were performed using three hiPSC lines (GiPSC, NCRM-5, WTC11), three Chiron concentrations (3, 6, 9 μM), and three seeding densities (60, 66, 72 x10^3^ cells/cm^2^; top). Image analysis studies were performed by imaging one hiPSC line (GiPSC) with one Chiron concentration (6 μM) at the beginning of differentiation across a range of seeding densities (bottom). Middle column: Output cell populations of all studies were analyzed by flow cytometry to identify percentage of cardiomyocytes by expression of cardiac troponin T. Right column: Cardiomyocyte purity was modeled as a function of seeding density and Chiron concentration (top). Phase contrast images of cells at the beginning of differentiation were correlated with cardiomyocyte purity through automated image analysis in Matlab (bottom).

**Fig 2:**
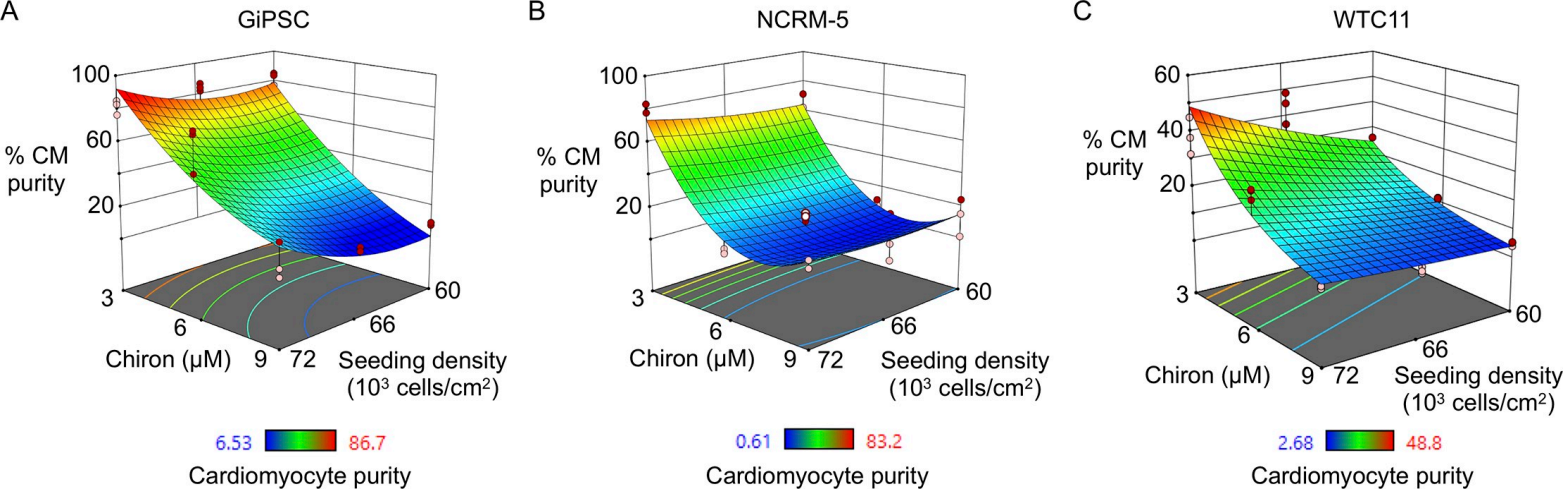
Chiron concentration and seeding density uniquely affect cardiac differentiation outcome. DOE (design of experiments) surface plots of directed differentiation outcomes in human induced pluripotent stem cell lines obtained from commercial (GiPSC; A), national bank (NCRM-5; B), and research institution (WTC11; C) sources. Red and pink circles indicate design points above and below predicted values, respectively. N = 9 per group per condition from 3 biological replicates (i.e., batches of hiPSC-cardiomyocytes).

In order to establish the relationship between Chiron concentration, seeding density, and cardiomyocyte purity, we used a design of experiments (DOE) approach to explore the experimental space for three hiPSC lines: GiPSC, NCRM-5, and WTC11 (Fig 1). HiPSCs were seeded at 60, 66, or 72 x10^3^ cells/cm^2^, and differentiation was started the following day at 24 hours with the application of 3, 6, or 9 μM Chiron. These ranges of values were chosen from development of in-house as well as published protocols [9,10,17]. Cells were dissociated into single cells and fixed at day 14 of differentiation, and cardiomyocyte purity was measured with flow cytometry using the cardiac marker cardiac troponin T (cTnT). DOE software was used to correlate differentiation outcomes with the nine different starting conditions with R^2^ values of 0.86 for GIPSC, 0.93 for NCRM-5, and 0.87 for WTC11 (N = 9, P<0.01, Fig 2). Importantly, the highest purity was attained with low, 3 μM Chiron in all hiPSC lines and declined with increased Chiron concentration, being significantly lower at 6 μM Chiron for NCRM-5 and at 9 μM for GiPSC and WTC11 hiPSCs at the highest seeding density. We observed large amounts of cell death and detachment between days 3–5 in high, 9 μM Chiron conditions in all lines, indicating a mismatch between cell density and Chiron concentration [10]. Only WTC11 hiPSCs demonstrate a strong dependence of purity upon seeding density at low Chiron concentration, suggesting inter-line variability appears when evaluating both Chiron concentration and seeding density. These findings demonstrate that across hiPSC lines the lowest Chiron concentration and highest seeding density maximizes cardiac purity.

### Thresholding of day 0 phase contrast images reveals optimal range of plate coverage

**Fig 3:**
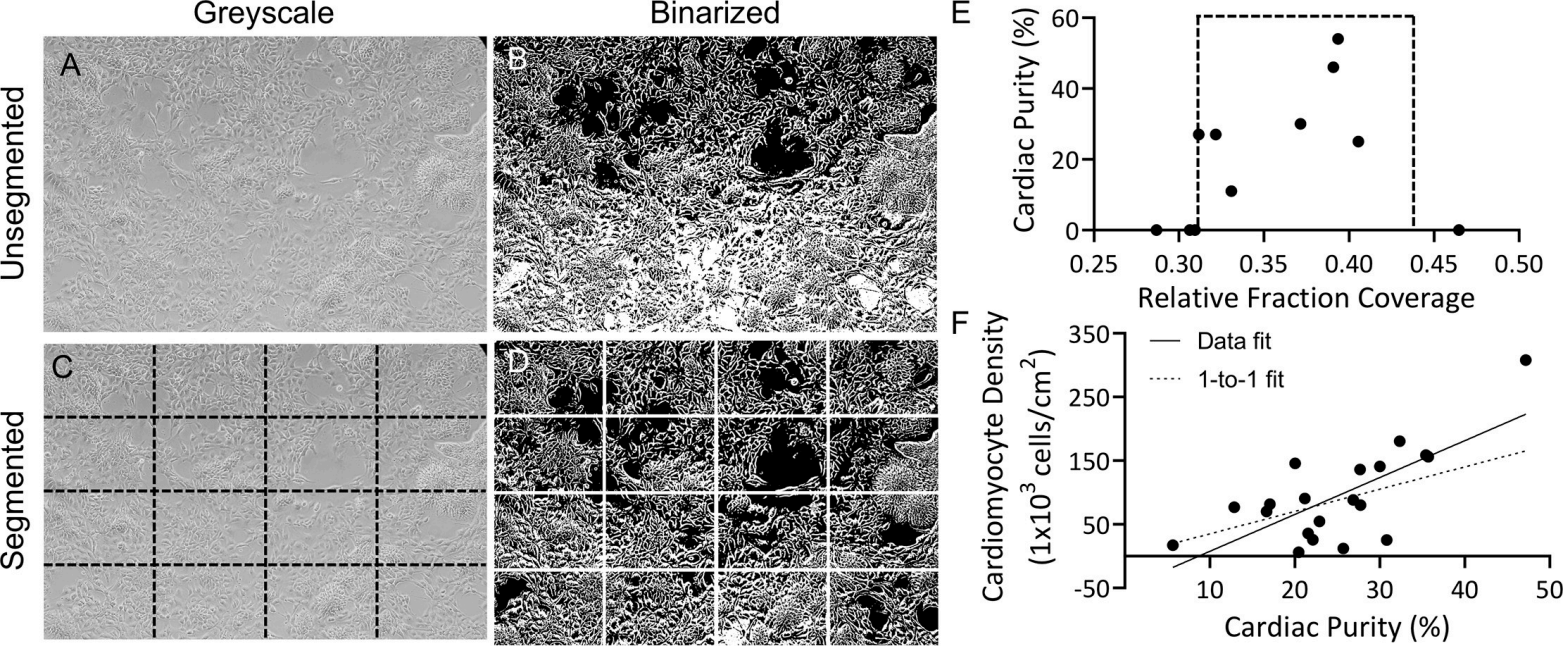
Segmented thresholding shows range of plate coverage for cardiomyocyte differentiation using 6 μM Chiron. (A) Brightfield image of Gibco hiPSCs (GiPSCs) at day 0 of differentiation. (B) Threshold of image without prior segmentation. (C) Segmented image, indicated by dashed black lines, and (D) reconstructed image after thresholding of segments. (E) GiPSC-cardiomyocyte purity plotted as a function of relative fraction coverage at day 0, dashed box indicated range of plate coverage in which differentiation runs produced cardiomyocytes. N = 11 independent differentiation runs; data points represent single differentiation run outcomes. (F) HiPSC-cardiomyocyte density per cm^2^ of growth area as a function of cardiac purity was fit with a linear regression. Data points represent single differentiation run outcomes (n = 20); solid line: linear regression, R^2^ = 0.51; dashed line: linear regression assuming 1-to-1 relationship between cardiomyocyte purity and density.

Because seeding density proved a critical factor in hiPSC-cardiomyocyte differentiation, we next sought to develop a robust and widely adaptable method to measure hiPSC density at the initiation of differentiation. Person-to-person variability in cell singularization and counting can greatly affect seeding density, so image analysis of day 0 hiPSCs was identified as a more uniform method to assess plate coverage. Phase contrast images of hiPSCs at day 0 from differentiation runs performed between the years 2015 and 2018 were used. Because GiPSCs with 6 μM Chiron was the most common and widely imaged in-house differentiation condition during that time period, analysis of this condition was pursued for these studies. This provided a data set of input images with enough variability to produce a robust analysis code. This concentration of Chiron is also widely reported in the literature [8–10,17,18]. Images were thresholded using a custom MATLAB code to determine a relative fraction of plate coverage (Fig 3A–3D). Brightfield images were used because of their ease of acquisition, yet unbiased, automated image analysis is challenging; namely, dark cell bodies are not included in the masked area, thus underestimating true cell confluency. Thus, we define a “relative fraction plate coverage” as a metric for confluency. These results were compared to flow cytometry data of cardiomyocyte purity after 14 days of differentiation to determine the optimal density range with 6 μM Chiron, which was chosen due to its widespread adoption (N = 11, Fig 3E) [8,10]. This analysis shows that a relative fraction plate coverage of 0.31 to 0.44 consistently produces successful differentiation runs, and that, in the case of 6 μM Chiron differentiation initiation, cell densities above or below this range yield little to no hiPSC-cardiomyocytes. To further underscore the power of predicting cardiac purity from the beginning of differentiation, we next determined the relationship between hiPSC-cardiomyocyte yield as a function of purity. Increasing cardiac purity resulted in a higher density of hiPSC-cardiomyocytes that exceeded the simple one-to-one scaling of cardiac purity. This was evidenced by a 1.7-fold increase in the slope of the linear regression fit to the data compared to the linear regression fit to a one-to-one scaling (N = 20, R^2^ = 0.51, Fig 3F). These results indicate that obtaining high purity is advantageous to limiting contaminant cells as well as maximizing cardiomyocyte yield.

### Lactate purification enriches hiPSC-cardiomyocytes and matures metabolic phenotype

**Fig 4:**
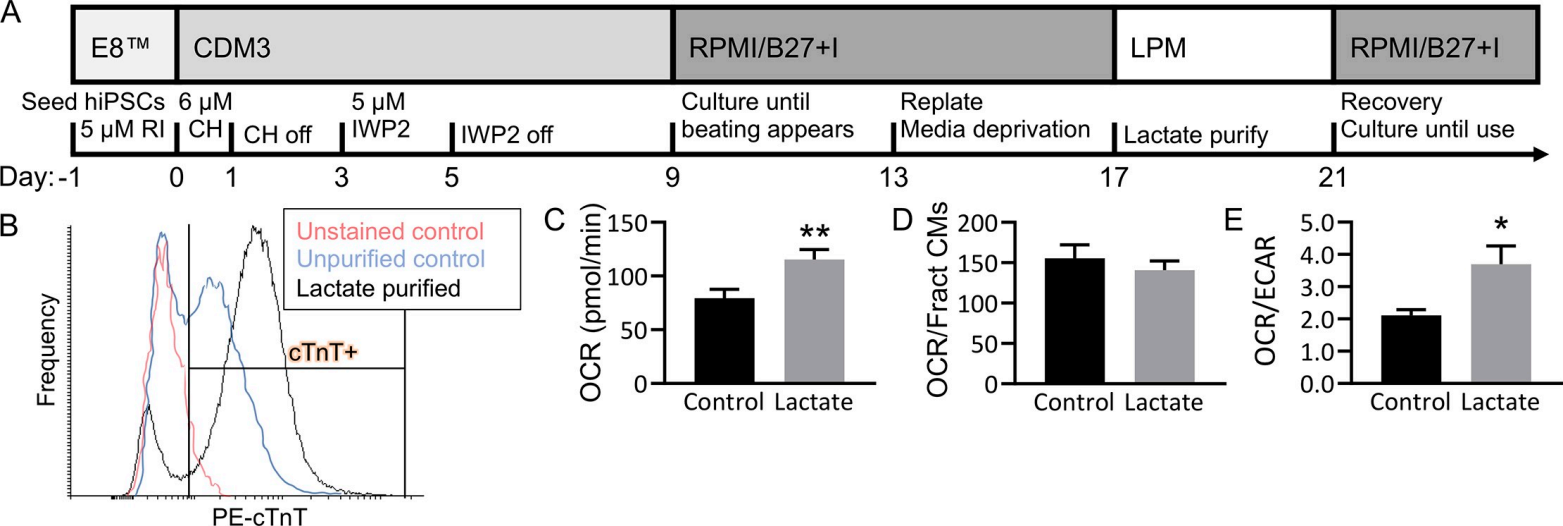
HiPSC-cardiomyocytes increase oxidative phosphorylation with lactate purification. (A) Cardiomyocytes were differentiated from Gibco hiPSCs (GiPSCs). (B) Control cells (blue, 51% cTnT+) were cultured for the same duration (with no starvation) and fed glucose medium, and lactate purified cells (black, 82% cTnT+) underwent glucose starvation and lactate purification from day 13 to 21 of culture. (C-E) Oxygen consumption rate (OCR; C & D) and extracellular acidification rate (ECAR), were measured at basal metabolic levels using a Seahorse XFe96 Analyzer, and values were normalized using a LIVE/DEAD stain. OCR was calculated independent of cardiac purity (C, **P<0.01) and normalized by cardiac purity (D, P = 0.46). (E) The ratio OCR/ECAR of Control (unpurified, 51% cTnT+) and Lactate (lactate purified, 82% cTnT+) samples was calculated (*P<0.05). N = 10 per group, bars represent mean ± SEM. (RI: rock inhibitor, CH: chiron, IWP2: inhibitor of wnt protein 2, CDM3: cardiac differentiation media 3, RPMI/B27 +I: RPMI/B27 with insulin, LPM: lactate purification media, cTnT: cardiac troponin T.).

The relative fraction of plate coverage determined with image analysis revealed a range for successful differentiation, but hiPSC-cardiomyocyte purity within this range varied from 15% to 60% purity. Previous work demonstrates that for optimal engineered cardiac tissue function, input hPSC-cardiomyocyte purity should roughly be between 40% and 75% [19]. In order to attain this level of purity, we investigated how the enrichment method of metabolic selection, or “lactate purification,” performed in our hands. We adapted the previously published protocol (Fig 4) [14] to fit our differentiation pipeline, to attain hiPSC-cardiomyocyte populations of >80% purity (Fig 4B).

During the lactate purification process, we observed increased spontaneous beating of lactate-treated hiPSC-cardiomyocytes. Because lactate purification relies on the unique metabolic versatility of cardiomyocytes, we hypothesized that the enrichment process changed the population-level metabolic phenotype of hiPSC-cardiomyocytes. To test this, we cultured unpurified cardiomyocytes (51% cTnT+) and lactate-purified cardiomyocytes (82% cTnT+) in parallel and measured their rates of oxygen consumption (OCR) and extracellular acidification (ECAR) with a Seahorse Xfe96 Analyzer. OCR increased by 1.5-fold in lactate-purified hiPSC-cardiomyocytes compared to unpurified control, indicating an overall increase in metabolic activity (n = 10, P<0.01, Fig 4C). Cardiomyocytes have a higher metabolic demand compared to non-cardiomyocytes [20], and thus the increase in OCR in purified samples is likely due to the increased proportion of cardiomyocytes being measured. To confirm test this hypothesis, we normalized OCR by cardiac purity and found no significant difference between unpurified and purified samples (Fig 4D, P = 0.46).

The ratio of OCR over ECAR increased by 1.8-fold in lactate-purified hiPSC-cardiomyocytes compared to the paired, unpurified control (n = 10, P<0.05, Fig 4E). The increase of this ratio indicates an increased metabolic reliance on oxidative phosphorylation (OXPHOS) versus glycolysis, a trademark of more metabolically mature cardiomyocytes.

Because this profiling technique provides population-level characterization, these results do not indicate single-cell metabolic shifts. However, results reveal a population-wide bioenergetic shift toward OXPHOS which reflects the metabolic behavior of developing cardiomyocytes. Taken together, these findings indicate that although lactate purification is an efficient means to enrich for hiPSC-cardiomyocytes, it significantly changes the bioenergetic phenotype of the output population.

### Engineered tissues form and function independently of purified cardiomyocytes

**Fig 5:**
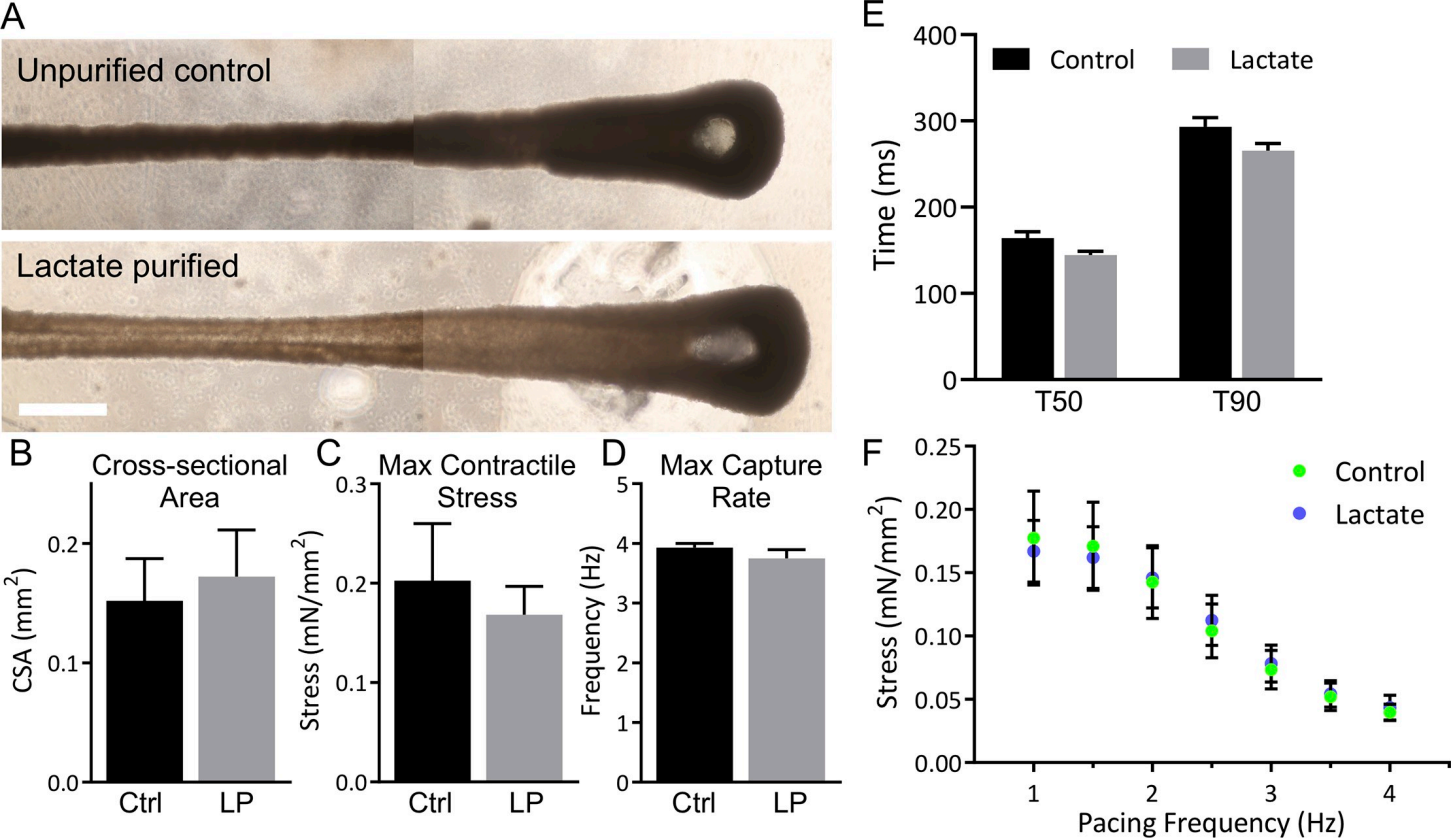
Lactate purified cardiomyocytes do not alter tissue formation via compaction and electromechanical function at one week. (A) Engineered cardiac tissues made with 5% human cardiac fibroblasts (hCFs) and unpurified hiPSC-cardiomyocytes (38% cTnT+; top) or lactate purified hiPSC-cardiomyocytes (82% cTnT+; bottom) were cultured for one week. Scale bar: 1 mm; images stitched together. (B-F) Control tissues (Ctrl, n = 8) and tissues with lactate purified cells (LP, n = 4) underwent mechanics testing at 1 week. Cross-sectional area (B), maximum twitch contraction stress (C), and maximum capture rate (D) were measured. (E) Relaxation time to 50% (T50) and 90% (T90) of maximum stress was measured. (F) Maximum stress production was measured at increasing pacing frequency. Data are represented as mean ± SEM.

To determine if the metabolic shift in lactate-purified hiPSC-cardiomyocytes affected functional output, we formed engineered cardiac tissues (ECTs) from purified (82% cTnT+, n = 4) or non-purified (38% cTnT+, n = 8) hiPSC-cardiomyocytes. In addition, 5% adult human cardiac fibroblasts (hCFs; passage 4) were included to aid tissue formation and function. ECTs were cultured for one week during which time all tissues formed and began to beat in syncytia (Fig 5A). At one week, tissues underwent mechanical testing to analyze functional performance. Cross-sectional area (CSA), a surrogate measure of tissues compaction, was not different between groups, which all compacted to approximately one third of initial tissue CSA (P = 0.73, Fig 5B). The force generated per cardiomyocyte during a maximum twitch contraction was not different between control (55 ± 36 pN/cardiomyocyte) versus lactate purification (34 ± 14 pN/cardiomyocyte; P = 0.30). Maximum twitch contraction stress (contractile force normalized by CSA) was measured under 1 Hz stimulation and did not differ significantly between groups (P = 0.70, Fig 5C).

The force kinetics of the tissues were also evaluated. The maximum capture rate, determined as the pacing frequency at which ECTs could capture at least 5 consecutive stimuli, was measured by pacing ECTs from 1 Hz to 4 Hz. ECTs showed no difference between groups (P = 0.24, Fig 5D). The time to 50% and 90% relaxation of peak force was compared at 1 Hz pacing and 30% stretch and did not significantly differ between groups (T50 P = 0.32, T90 P = 0.12, Fig 5E). Finally, force production as a function of pacing frequency was measured between 1 Hz and 4 Hz. Tissues of both groups demonstrated a negative force-frequency relationship (FFR) and were not significantly different at any pacing frequency (P>0.99 at all frequencies, Fig 5F). These results suggest that the metabolic shift resulting from lactate purification is not reflected in engineered cardiac tissue performance at one week after tissue formation.

## Discussion

Nuances and variability in cardiomyocyte differentiation and purification from human pluripotent stem cells has challenged widespread adoption. In this study, we optimize differentiation conditions and investigate the functional implications of hiPSC-cardiomyocyte enrichment through metabolic selection. Our results show that (1) cell seeding density and Chiron concentration impact cardiomyocyte differentiation in a similar way across hiPSC lines, with a global optimum of 3 μM Chiron and high (72 x 10^3^ cells/cm^2^) seeding density in the presence of albumin, but that the resulting range of hiPSC-cardiomyocyte purity is unique to each line; (2) analysis of phase-contrast images taken at day 0 of differentiation gives a range of plate coverage values to successfully differentiate hiPSC-cardiomyocytes; (3) purification with lactate-based medium enriches for hiPSC-cardiomyocytes while shifting the population’s metabolic phenotype towards oxidative phosphorylation; and (4) the metabolic shift in lactate-purified cardiomyocytes does not significantly improve engineered cardiac tissue force production under these conditions after one week of culture in 3D tissues.

For cardiomyocyte differentiation to be successful, the mesodermal lineage must first be induced in hiPSCs, a process achieved through the application of Chiron in the protocol used for these experiments [10]. Laco *et al*. recently demonstrated that the efficacy and optimal concentration of Chiron in hPSC-cardiomyocyte differentiation depends on the proportion of the hPSC population in the S/G2/M phase of the cell cycle [11]. Additionally, the group showed that the confluency of hPSCs in culture correlates with the distribution of cell cycle stages in the population. Our results suggest that, across three hiPSC lines, low (3 μM) Chiron concentrations with higher seeding density groups performed better than lower seeding density at any higher Chiron concentration. This is in agreement with findings from Laco et al. that 3 μM Chiron in low S/G2/M populations (high density) produced roughly 40% pure hESC-cardiomyocyte populations while in high S/G2/M populations (low density), produced <5% pure hESC-cardiomyocyte populations [11]. Interestingly, the study reports the biggest difference in differentiation outcomes based on both Chiron concentration and S/G2/M levels between 5 μM and 6 μM Chiron. Although other factors such as dose/timing of Wnt inhibition and substrate coating impact the differentiation process [8], our data (Fig 2) clearly demonstrates the dramatic range of cardiomyocyte purities that result from only altering two: Chiron concentration and seeding density.

It is unsurprising that, though we observed similar trends across three hiPSC lines, differences in differentiation outcomes were present. Recent work has shown that different hiPSC lines possess distinct transcription profiles and that these profiles are correlated with more or less cardiogenic hiPSC lines [21,22]. This may explain why some lines show more sensitivity to Wnt activation via Chiron compared to others. Additional transcriptomic studies have demonstrated that within hiPSC lines there is heterogeneity in both pluripotency and overall transcript expression [6,23]. This adds yet another source of variability to hiPSC-cardiomyocyte differentiation. For practical adoption and optimization of cardiomyocyte differentiation techniques, genetic profiling is not feasible, and we propose using hiPSC colony morphological assessment (dense interiors and clean borders) and image analysis techniques early during differentiation to reduce variability during differentiation.

In order to reliably and repeatably perform successful differentiation runs, we developed an easily adoptable image analysis protocol to determine relative plate coverage from brightfield phase-contrast images to validate a range of cell densities that successfully yields hiPSC-cardiomyocytes (Fig 3E) and recapitulates the sloped relationship of the DOE analysis (Fig 2A). In order to obtain a large image data set, one of the most commonly used in-house differentiation conditions, namely GiPSCs with 6 μM Chiron, was used. Notably, differentiation yields zero cardiomyocytes outside of this range set by our seeding density followed by 24 hrs culture prior to Chiron application (Fig 3E). By determining the fraction coverage necessary for a range of Chiron concentrations to produce hiPSC-cardiomyocytes, the method can then be reversed, and, given a cell density, Chiron can be applied at the appropriate time to ensure successful hiPSC-cardiomyocyte differentiation or culture time extended to reach an optimal plate coverage. This methodology could thus potentially eliminate one of the greatest sources of variability in the differentiation process.

High purity hiPSC-cardiomyocyte populations of greater than 80% are currently considered the standard for clinical translation [24], which necessitates the use of enrichment protocols for most differentiation runs. These methods take the form of genetic selection [25], antibody-based selection [26], and most commonly, metabolic selection [14]. However, little is known about if and how these enrichment processes select for specific hiPSC-cardiomyocyte phenotypes or sub-populations. We confirm that, in the case of lactate purification, the enriched cell population shifts towards a higher dependence on oxidative phosphorylation and show that oxygen consumption rate per cardiomyocyte does not vary. Within one post-natal week, human fetal cardiomyocytes shift from mostly glycolysis to oxidative phosphorylation due to increased metabolic demand [27], and hPSC-cardiomyocytes have been shown (similar to fetal cardiomyocytes) to utilize mostly glycolysis for energy production [28,29]. The high expression level of HIF1α in hPSC-cardiomyocytes has been implicated in their immature metabolic phenotype, and culture in glucose-deprived media reduces HIF1α expression [28]. This suggests that the metabolic stress induced during metabolic selection is responsible for the shift toward oxidative phosphorylation in our lactate-purified hiPSC-cardiomyocytes.

The excitation-contraction-metabolism axis in cardiomyocytes is critical for a mature phenotype, and for effective therapeutics, cardiomyocyte electromechanical function is critically important [30]. To determine how increased oxidative phosphorylation and purity of cardiomyocytes impacted immediate functional output, we compared contractile force of engineered cardiac tissues composed of unpurified or purified cardiomyocytes. Because ECTs reach peak performance with 40%-70% cardiomyocyte purity [19], we included 5% adult human cardiac fibroblasts. We observed no significant differences in tissue compaction, maximum contractile twitch stress, or maximum capture rate, which may be explained by multiple factors. Firstly, metabolic function is not a surrogate measure for electromechanical function. The conditions, namely glucose-deprived culture medium used for metabolic maturation in lactate-purified hiPSC-cardiomyocytes did not include stimulants for electromechanical maturation. Secondly, both unpurified and purified cardiomyocyte-containing ECTs were subjected to the same static uniaxial stress and 1 Hz electrical pacing during the one week of culture time, which may not be enough time or biomechanical stimulation for growth/hypertrophy [19,31,32]. Thirdly, because ECT electromechanical function is closely linked to cardiomyocyte purity [19,33], the purity of control and lactate purified ECTs, which fall on either side of the ideal cardiomyocyte purity of roughly 50–60% [19], may mask changes resulting from cardiomyocyte bioenergetic phenotype. Finally, after lactate purification, tissues were returned to a glucose-based medium and lacked fatty acid supplementation, which has recently been shown to support electromechanical maturation [16,34]. Our findings suggest that though lactate purification changes hiPSC-cardiomyocyte metabolic phenotype, a corresponding matured electromechanical phenotype is not present after short culture periods (1 week) in standard glucose-containing medium (RPMI/B27). We hypothesize that continually driving oxidative phosphorylation metabolism, potentially via lactate or fatty acid supplementation [34] will further enhance the excitation-contraction-metabolism axis for greater force production and kinetics.

In summary, this study provides insights for a broader and more consistent adoption of hPSC-cardiomyocyte differentiation and purification for translational applications. By harnessing the power of DOE and automated image analysis, we develop optimized differentiation models for multiple hiPSC lines, suggest low Chiron (3 μM) and a moderately-high cell seeding density provide highest cardiac purity when directed differentiation is initiated 24 hours after cell seeding, and provide a practical means to implement these models at the bench. As hPSC-cardiomyocyte therapies reach clinical translation, it is critical for the cardiovascular engineering field to pursue reproducible, transparent means of generating high purity cell populations with well-defined phenotypes. The findings presented here provide important tools and advances to achieve that goal.
